# Supplementary material for: A normalized drug response metric improves accuracy and consistency of anticancer drug sensitivity quantification in cell-based screening
Source: Commun Biol. 2020 Jan 23;3:42. doi: 10.1038/s42003-020-0765-z (PMC6978361; doi:10.1038/s42003-020-0765-z)
Supplement: Supplementary file 6 — Description of Additional Supplementary Files [file 42003_2020_765_MOESM6_ESM.docx]

**Description of Supplementary data**

**Supplementary Data 1**

List of compounds tested in the study. The generic names of 131 compounds along with their nonproprietary Name, mechanism of action, FDA clinical phase/approval status, supplier, supplier reference code, lowest and highest concentration tested are tabulated in this data file.

**Supplementary Data 2**

List of cell lines used in the study. The names of cell lines, tissue type, source & source code, growth medium, cell seeding amount per well (384 well plate) and doubling times used in the experiments of this study are provided in the data file.

**Supplementary Data 3**

Data for drugs effect classification in MDA-MB-361 cell line. The names of 131 drugs, their mechanism of actions and their effects (classified as lethal, sub-effective, non-effective & growth-stimulatory) are incorporated in the data file.

**Supplementary Data 4**

Raw luminescence readouts of screened drugs. The raw data of luminescence readouts for 131 drugs screened against MCF-7, MDA-MB-231, MDA-MB-361, HDQ-P1 and MIA-Paca-2 cell lines are provided. Furthermore, the data of replicated screen for MCF-7 is also incorporated in the files. The luminescence was measured using a PHERAstar FS plate reader (BMG Labtech).
