# Supplementary material for: A normalized drug response metric improves accuracy and consistency of anticancer drug sensitivity quantification in cell-based screening
Source: Commun Biol. 2020 Jan 23;3:42. doi: 10.1038/s42003-020-0765-z (PMC6978361; doi:10.1038/s42003-020-0765-z)
Supplement: Supplementary file 8 — Peer Review File [file 42003_2020_765_MOESM8_ESM.pdf]

***Editorial Note:*** *This manuscript has been previously reviewed at another Nature Research journal. The manuscript was considered suitable for publication without further review at Communications Biology.*
